# Measuring Physical Health in Patients on Antipsychotic Medications

**DOI:** 10.1192/bjo.2024.349

**Published:** 2024-08-01

**Authors:** Elizabeth Beavis, Jessica Morris, Katherine McMenzie

**Affiliations:** 1 Hitchin, United Kingdom; 2 Northumbria Healthcare, Newcastle Upon Tyne, United Kingdom

## Abstract

**Aims:**

Mental illness is associated with poorer physical health and reduced life expectancy in comparison to the general population. This is influenced by many factors, one of which is medication related. Antipsychotics can have multi system effects on the body such as increasing the risk of metabolic syndrome and cardiovascular disease. Our objective was to understand current challenges when monitoring patients' physical health and thereby improve overall health outcomes.

**Methods:**

Utilising a clinical audit template, the study group was 9 inpatients during cycle 1 and 10 inpatients during cycle 2, who were prescribed antipsychotics on an Old Age Psychiatry ward. Northumberland, Tyne and Wear (NTW) antipsychotic monitoring guidelines were used as criteria which stipulate that blood tests, ECGs, BMI, waist circumference, side effects and lifestyle effects should be recorded at defined intervals. A proforma highlighting these guidelines was created following audit cycle 1 and utilised by the MDT on the ward, the purpose of cycle 2 was to compare findings following the implementation of the proforma. The standard to meet was that 100% of patients should fulfil the guidelines. Data was collected by retrospectively reviewing paper and electronic notes.

**Results:**

Audit cycle 1 revealed 0 of the patients met the physical health criteria. 0 had the full set of required bloods in the correct timeframe, 0 had waist circumference checked and 2 and 1 patients had side effect and lifestyle effects documented respectively. By comparison, ECGs and BMIs were recorded well. Audit cycle 2 demonstrated significant improvement in all areas. 9 patients had bloods accurately measured. 3 and 6 had side effect and lifestyle reviews respectively. ECGs and BMIs continued to be monitored well. However, waist circumference remained poor with 1 patient recorded. Qualitative feedback when presenting these findings to the MDT highlighted an interest debate into the cost/benefit of measuring waist circumference with the main point being not wishing to cause undue anxiety to the patient.

**Conclusion:**

The use of an accessible proforma clearly outlining the criteria to meet for each patient proved valuable in improving the monitoring of physical health parameters. This study highlighted a need for increased awareness of metabolic syndrome and the importance of empowering patients with knowledge regarding their healthcare to help tailor a patient-centred approach to physical health monitoring. Our presentation aims to encourage discussion among attendees around measuring waist circumference and raise awareness of metabolic syndrome.

